# Primary tumor- and metastasis-derived colon cancer cells differently modulate connexin expression and function in human capillary endothelial cells

**DOI:** 10.18632/oncotarget.4894

**Published:** 2015-08-06

**Authors:** Dominique Thuringer, Kevin Berthenet, Laurent Cronier, Eric Solary, Carmen Garrido

**Affiliations:** ^1^ INSERM, U866 Faculty of Medecine, 21000 Dijon, France; ^2^ University of Bourgogne-Franche-Comté, 21000 Dijon, France; ^3^ CNRS ERL7368, STIM Lab, University of Poitiers, 86022 Poitiers, France; ^4^ INSERM, U1170, Institut Gustave Roussy, 94508 Villejuif, France; ^5^ CGFL, 21000 Dijon, France

**Keywords:** GJIC, Cx32, Cx43, HSP27, CXCR2

## Abstract

A gradual loss of functional gap junction between tumor cells has been reported with colorectal cancer (CRC) progression. Here, we explored if colon cancer cells could also affect gap junctions in blood capillary cells. Human microvascular endothelial cells (HMEC) were cultured with two CRC cell lines established from a unique patient. SW480 cells, derived from the primary tumor, migrate much faster across HMEC monolayer than SW620 cells derived from a metastatic site. The motile SW480 cells highly express and release HSP27 that increases gap junction formation with HMEC. Soluble HSP27 phosphorylates the connexin Cx43 on serine residues and induces its interaction with the oncoprotein 14-3-3, which promotes Cx43 delivery at the plasma membrane. The factors secreted by less motile SW620 cells do not affect Cx43 expression but up-regulate the expression of the connexin Cx32 through an activation of the chemokine receptor CXCR2. In turn, SW620 secreted factors induce tubulogenesis and ATP release. Altogether, cell lines derived from CRC primary tumor and metastasis differentially adapt endothelial cell functions by modulating connexin expression through released mediators.

## INTRODUCTION

The outcome of patients who develop a metastatic colorectal carcinoma (CRC) remains poor, emphasizing the need to better understand the mechanisms of disease progression and metastatic dissemination [1–3]. During metastatic dissemination, a cancer cell quits the primary tumor to enter capillaries of the blood system (intravasation), translocates through the bloodstream to capillaries of distant tissues, exits from the bloodstream (extravasation) across the microvascular endothelium, and finally adapts to the foreign microenvironment of these tissues to proliferate and form new tumor foci. In this process, interactions between cancer cells and microvascular endothelial cells are of utmost importance.

A disturbance of gap junction intercellular communication (GJIC) has been involved in both primary tumor formation [4, 5] and progression toward metastasis [6, 7]. Gap junctions are specific cell-to-cell channels formed by integral membrane proteins called connexins (Cx). These junctions play a role in cell growth and differentiation and in tissue homeostasis [8, 9]. In addition to forming channels that enable a direct exchange of ions and small molecules between cells, Cx are involved in transcription regulation [10, 11]. The most widely studied Cx is connexin 43 (Cx43) [12], which is frequently down-regulated in human tumors, *e.g*. Cx43 loss was associated with cancer progression [13]. The redistribution of Cx from the plasma membrane to intracellular compartments is another feature of cancer cells, as described for Cx32 and Cx43 during CRC development [11].

The effects of CRC cells on Cx expressed in endothelial cells is less known. Here, we explore how CRC cells modulate Cx-expression and function in endothelial cells. For that purpose, we use two human CRC cell lines established from the same patient [14]. SW480 cell line was established from the primary tumor whereas SW620 was derived from a metastatic site [15]. We show that the culture medium of these cell lines have distinct effects on human microvascular endothelial cells. SW480 cells secrete the small heat shock protein HSP27 (also called HSPB1) that promotes Cx43 phosphorylation and the formation of intercellular gap junctions with HMEC. SW620 cells secrete interleukin-8 (IL-8) and promote receptor CXCR2 expression in HMEC; in turn, CXCR2 increases Cx32 expression and induces ATP release. Such distinct effects could account for the differential ability of cancer cells to migrate through the endothelium, to form metastases and to develop new tumor foci in distant organs.

## RESULTS

### HSP27 favors communication between endothelial and cancer cells

**Figure 1:**
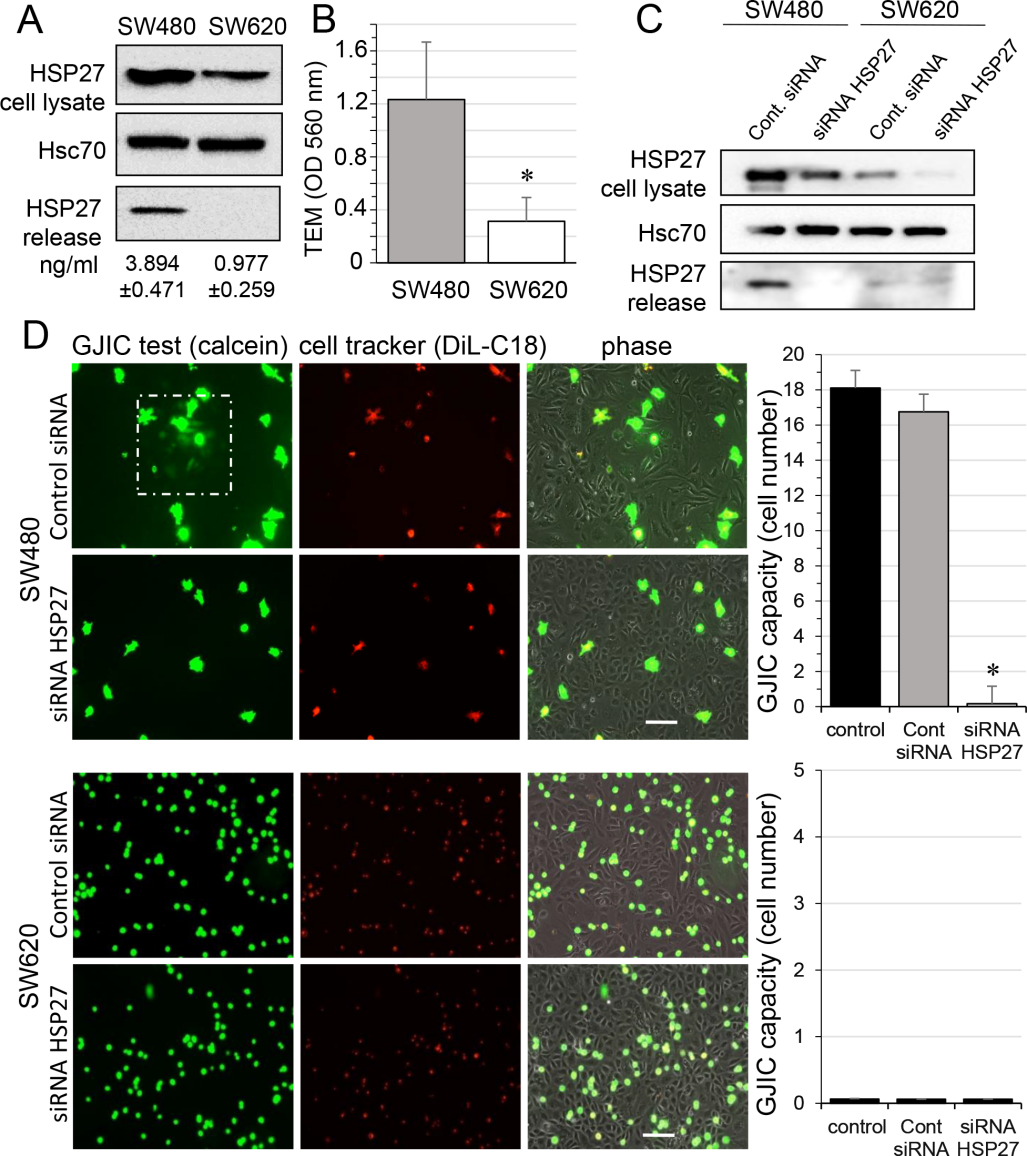
HSP27 knockdown inhibits the gap junctional coupling between SW480 cells and HMEC **A.** Expression of HSP27 in the two CRC cell lines, SW480 and SW620 cells. Detectable amounts of HSP27 in supernatants of SW480 cells but not of SW620 cells (media collected after 12 h). Immunoblots representative of 5 experiments (Hsc70 as loading control). Values indicate amounts of HSP27 measured by ELISA in supernatant of SW480 and SW620 cells for 12 h (mean ± SD; *n* = 4; *P*-values < 0.01). **B.** Transendothelial migration (TEM) of CRC cell lines. Control HMEC monolayers grown on Transwells were kept in FCS-free conditions overnight. Untreated SW480 and SW620 cells (3 × 10^5^) were added into the wells. After coculturing for 6 h, invasive cells on the membrane bottom were stained and quantified at OD 560 nm after extraction (mean ± SD; *P-values < 0.01; *n* = 5). **C.** siRNA transfection decreases the HSP27 expression in CRC cells and suppressed its release by SW480 cells. Representative immune-blot of HSP27 protein level in both SW480 and SW620 cells transfected with control siRNA or siRNA HSP27 for 2 days (*n* = 5; Hsc70 as loading control). **D.** Functional GJIC between SW480 cells and HMEC. The both CRC cell lines, SW480 and SW620 cells (donors), were preloaded with calcein/AM and DiL-C18. Calcein diffuses through gap junctions, while DiL-C18 does not. Labelled CRC cells are then plated with unlabeled HMEC monolayer (receivers). HMEC establishing GJIC with CRC cells become fluorescent by calcein diffusion. Only SW480 cells establish GJIC with HMEC and siRNA HSP27-transfected cells improved it (upper panels). No calcein diffusion was observed from SW620 cells in spite of their adhesion to HMEC (lower panels). Phase-contrast microphotographs after 6 h of culture (representative of 6 experiments; Bar 100 μm). Right, histogram represents the total cell number of HMEC receiving dye (calcein) per CRC cell (mean ±SD, *n* = 3; *P-values < 0.01 vs control).

We first confirmed previous reports [16, 17] showing that the small heat shock protein HSP27 was more expressed in, and secreted by, primary tumor-derived SW480 cancer cells when compared to their metastasis-derived counterpart SW620 cells (Fig. 1A). We also noticed that SW480 cells moved much faster than SW620 cells across a human endothelial cell monolayer (Fig. 1B). Looking for a link between these two observations, we used a specific siRNA to decrease the expression of HSP27 in the two cell lines, and to suppress its secretion by SW480 cells (Fig. 1C). These cells were subsequently double loaded with calcein, a dye that passes through gap junctions, and DiL, a membrane-bound dye (Fig. 1D; [18]). The labelled CRC cells were co-cultured with unlabeled HMECs for 6 hours. Although both SW480 and SW620 cells adhered to the endothelial monolayer, the calcein transfer attesting the formation of GJIC was observed only with SW480 cells. HSP27 down-regulation did not affect cancer cell adhesion to endothelial cells, but abolished the calcein transfer from SW480 to HMECs. A similar result was obtained by inhibiting HSP27 expression in SW480 cells with the OGX427 antisense oligonucleotide and was partially antagonised by the concomitant addition of recombinant human HSP27 (rhHSP27) to the culture medium (Suppl. Fig. S1). These results suggest that HSP27 secreted by SW480 cells increases the communication between cancer cells and endothelial cells.

### Extracellular HSP27 also promotes the communication between endothelial cells

**Figure 2:**
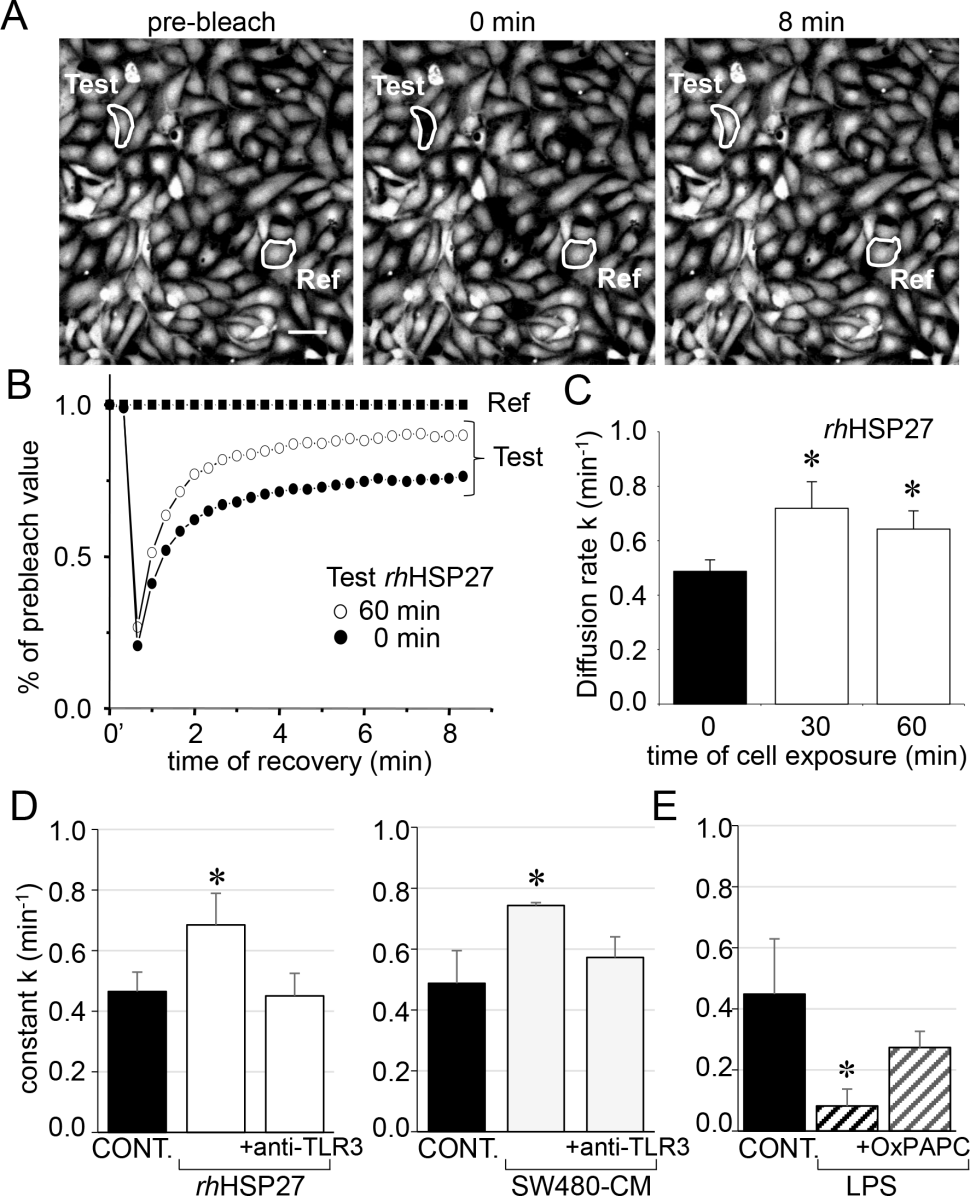
Extracellular HSP27 increases the endothelial gap-junction coupling **A.** FRAP analysis of cell-to-cell communication. Digital images of fluorescence distribution in a HMEC monolayer at three times during a typical gap-FRAP experiment: prebleach, just after bleaching (0 min) and after fluorescence recovery (8 min). Bars 20 μm. Corresponding fluorescence intensities (% of prebleach value) versus time in tested cells. The fluorescence in one unbleached cell (Ref) was used to correct the artefact loss of fluorescence. Note the fluorescence recovery follows an exponential time course when the bleached cells (circles) are interconnected by open gap-junction channels to unbleached cells (black squares are Ref). The relative permeability of gaps is given by the time constant *k*. **B.** Recombinant human HSP27 (rhHSP27) effect on GJIC. Graph represents mean ± SEM of the fluorescence redistribution after photobleaching in coupled HMEC in control (●) or after 60 min (○) with rhHSP27 (5 μg/ml). **C.** Histogram shows *k* values measured after the rhHSP27 addition for 0, 30 and 60 min (mean ± SD, *n* = 8; *P < 0.05 *vs* control [*t* = 0 min]). **D.** Both rhHSP27 and SW480-conditioned media (-CM; collected after 6 h) increase the GJIC in a TLR3-dependent manner. Cells exposure for 30 min, in the absence or the presence of neutralizing anti-TLR3 antibody (20 μg/ml) (mean ± SD, *n* = 4; *P < 0.01 *vs* control). **E.** LPS (1 μM) blocks GJIC within 60 min. This inhibitory effect was prevented by OxPAPC (30 μg/ml), a TLR4/TLR2 inhibitor (mean ± SD, *n* = 4; *P < 0.01 *vs* control).

To analyze the effects of rhHSP27 on gap junctions between HMECs in confluent monolayers, we used the gap-FRAP technique [18]. Briefly, HMECs were loaded with a diffusible tracer (calcein/AM) before suppressing their fluorescence with a laser beam, then measuring the fluorescence recovery resulting from the intercellular diffusion of calcein. Fig. 2A shows typical changes in the fluorescence of cell after photobleaching. Addition of rhHSP27 to the culture medium (5 μg/ml [19], open circles) increased the fluorescence recovery after photobleaching when compared to controls (Fig. 2B). The amount of HSP27 secreted by SW480 cells seems very low compared with the exogenously added in HMEC cultures (Suppl. Fig. S1). However this was a dosage for the whole fluid bathing the cells whereas the secretion by SW480 cells must be considered in their closed vicinity near the endothelial cell. Cells must be adherent to establish gap junction channels (the intercellular space ranges between 2 and 4 nm). So the real quantity of HSP27 secreted by the SW480 and collected by the endothelial cell is certainly much higher that the dose measured (diluted) in the whole bath. The diffusion rate constant *k* (min^−1^), which is an index of gap junction permeability, increased within 30 min from 0.487 ± 0.042 min^−1^ in untreated cells to 0.719 ± 0.097 min^−1^ in rhHSP27- treated cells (mean ± SD, *n* = 8), then slowly decreased (0.642 ± 0.066 min^−1^ after 1 hour, Fig. 2C). This effect of rhHSP27 was prevented by pretreating the cells with a neutralizing antibody against Toll-Like Receptor-3 (anti-TLR3 mAb 20 μg/ml) for 1 h (Fig. 2D, left panel; [19]). A similar result was obtained by incubating HMEC with SW480 cell-conditioned medium (SW480-CM; collected after 6 h in culture), i.e. the *k* value increased in a TLR3-dependent manner (Fig. 2D, right panel). Conversely, LPS (1 μM) decreased *k* value, an effect prevented by the TLR4 inhibitor OxPAPC (30 μg/ ml) (Fig. 2E). Altogether, these results indicate that soluble HSP27 increases the communication between neighboring cells.

### SW480-CM promotes the phosphorylation of Cx43 in endothelial cells

**Figure 3:**
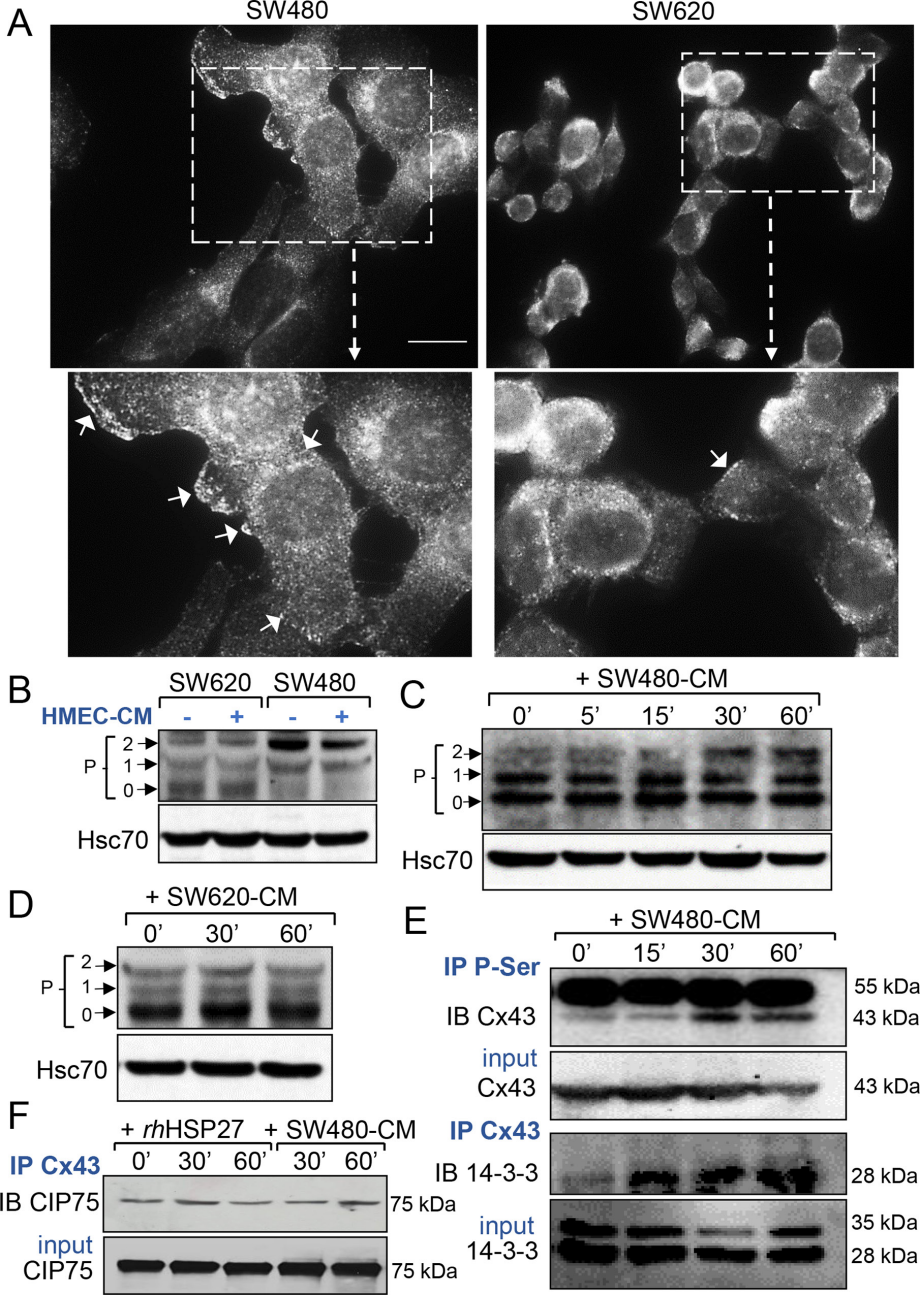
Phosphorylation at serine sites of endothelial Cx43 and 14-3-3 binding characterize the SW480-CM-induced GJIC increase **A.** Immunofluorescence detection of Cx43 in SW480 cells and SW620 cells (Bar 20 μm). The dotted areas are enlarged in the inserts below. Arrows indicated the Cx43 plaques at the plasma membrane in cells. Representative of 5 experiments. **B.** Western blot of Cx43 in whole cell lysates from SW480 and SW620 cells, exposed or not the HMEC-conditioned media (-CM, collected after 6 h). P0, P1 and P2 denote the three major Cx43 migration bands (Hsc70 as loading control). **C.** Time-dependent increase in Cx43 phosphorylation induced by the SW480-CM in confluent HMEC. Whole cell lysates in HMEC exposed to SW480-CM (collected after 6 h) for time periods as indicated (Hsc70 as loading control; representative of 3 experiments). **D.** No change in the phosphorylation state of the endothelial Cx43 was induced by the SW620-CM (*n* = 3). **E.** Serine phosphorylation and Cx43 immuno-precipitate in HMEC exposed to SW480-CM. Note that the Cx43 interaction with the protein 14–3-3 precedes its phosphorylation in serine sites. Data are representative of 3 independent experiments. IP, immunoprecipitation; IB, immunoblot; Input material, total amount of proteins per lane. IgG heavy chain at 55 kDa. **F.** Neither rhHSP27 nor SW480-CM affect the low amount of CIP75 interacting with Cx43 (representative of 3 experiments).

Immunofluorescence analyses detected Cx43 mainly at the surface of SW480 cells and in the cytoplasm of SW620 cells (Fig. 3A). The diffusion of calcein between cells depends on the opening of gap junction channels present at the plasma membrane of adherent cells. Since the formation of functional Cx43 gap junction channels requires connexin phosphorylation [20–22], we performed immunoblot analyses of whole-cell extracts using a rabbit polyclonal antibody that recognizes several forms of the phosphorylated protein [12, 18, 21, 22]. SW480 and SW620 cells expressed distinct patterns of Cx43 (Fig. 3B). SW480 cells expressed mainly a phosphorylated form of Cx43 (called P2 on Fig. 3B), as confirmed by immunoblot treatment with alkaline phosphatase (Suppl. Fig. S2A), whereas SW620 cells expressed mostly the unphosphorylated protein (called P0 on Fig. 3B). Addition of HMEC-CM did not have any effect on the pattern of Cx43 expression in these two cancer cell lines (Fig. 3B and Suppl. Fig. S2A). In confluent endothelial cells, Cx43 was detected mainly as P0 and P1 forms. Incubation of these cells with SW480-CM induced the expression of the phosphorylated P2 isoform (Fig. 3C and Suppl. Fig. S2), which was not observed when HMECs were cultured with SW620-CM (Fig. 3D). The phosphorylation status of Cx43 in HMEC is further demonstrated in Suppl. Fig. S2. Immunoprecipitation of serine-phosphorylated proteins followed by immunoblotting with an anti-Cx43 antibody demonstrated that Cx43 was phosphorylated on serine residues in HMECs upon incubation with SW480-CM (Fig. 3E, upper panels). Looking for the consequences of Cx43 phosphorylation, we immunoprecipitated Cx43, then looked for interaction either with 14-3-3, which was shown to regulate the assembly of Cx43 multimers and their incorporation into existing gap junctional plaques [23, 24], or with CIP75 (Ubiquitine-like-Ubiquitine-associated protein), which regulates Cx43 proteolytic degradation [25, 26]. Incubation of HMECs with SW480-CM promoted the recruitment of 14-3-3 to Cx43 (Fig. 3E, lower panels) while having no effect on Cx43 interaction with CIP75 (Fig. 3F). Of not, rhHSP27 addition to HMEC culture medium also failed to increase Cx43 interaction with CIP75 (Fig. 3F). Moreover, we did not detect a specific ubiquitination of Cx43 in the tested conditions (Suppl. Fig. S2C). Thus, SW480-CM or rhHSP27 did not target Cx43 for proteasomal degradation. Altogether, our results suggest that SW480-CM induces the phosphorylation of Cx43 on serine residues and the subsequent binding of 14-3-3, enhancing the GJIC between cells [23, 24].

### SW620-CM induces the expression of a functional Cx32 hemi-channel in endothelial cells

**Figure 4:**
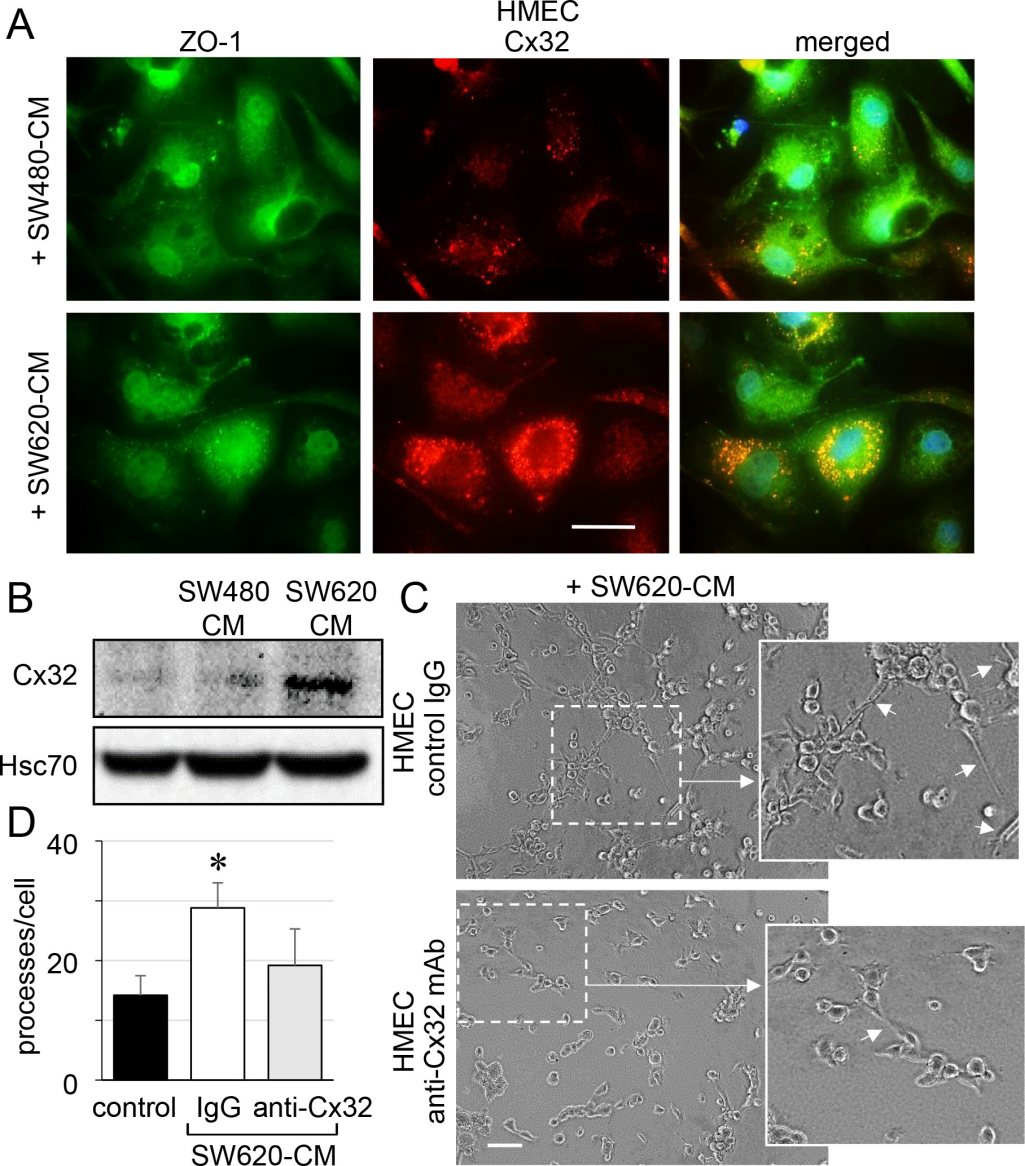
SW620 cell-secreted factors overexpress the endothelial Cx32 favoring tubulogenesis **A.** Endothelial cell localization of Cx32 in CRC cell-conditioned media. HMEC were stimulated with SW480-CM or SW620-CM for 6 h and double-stained for ZO-1 and Cx32. Representative micrographs showing the strong labelling of Cx32 induced by SW620-CM and the combined image of co-localization with ZO-1 (yellow); DAPI staining of nuclei (*n* = 3, bar 20 μm). **B.** SW620-CM increase the Cx32 expression in HMEC. A higher Cx32 protein level was detected in response to SW620-CM compared with SW480-CM by immune-blot analysis (no cell expression in unstimulated HMEC). Representative of 5 experiments (Hsc70 as loading control; 150 μg/lane). **C-D.** *In vitro* tubulogenesis assay of HMEC pretreated or not (control IgG) with inhibitory monoclonal antibody against Cx32 (anti-Cx32 mAb). HMEC were plated on Matrigel-coated 24-well plates, incubated with SW620-CM for 6 h, and photographed. **C.** Representative photos of tube formation in HMEC intracellularly delivered with 0.2 μg anti-Cx32 mAb or control IgG (Bar 80 μm). The dotted areas are enlarged in the inserts on the right. Arrows indicated branch points. **D.** Number of branch points per field of view was quantified (at least 80 single cells were scored; mean ± SD, *n* = 4; *P < 0.01 *vs* control).

Immunofluorescence analyses revealed that unstimulated HMEC expressed very low levels of Cx32 (not shown) and that the protein was only weakly expressed at the apical membrane of some cells after 6 h of exposure to SW480-CM (Fig. 4A). In contrast, we detected a strong apical membrane and cytoplasmic expression of Cx32 in HMEC exposed to SW620-CM for 6 hours (Fig. 4A). Immunoblot analysis of cell lysates identified a drastic increase in Cx32 expression in HMEC exposed to SW620-CM (Fig. 4B). Using an *in vitro* matrigel tube formation assay [19], we observed also that SW620-CM could promote the ability of endothelial cells to form capillary-like structures (i.e., increased branches per cell; Fig. 4C, 4D). To explore the contribution of Cx32 to this effect, we performed loss-of-function experiments through intracellular transfer of a Cx32 blocking monoclonal antibody [27]. Cx32 blockade dramatically reduced the ability of HMEC incubated with SW620-CM to form capillary-like structures (Fig. 4C, 4D).

**Figure 5:**
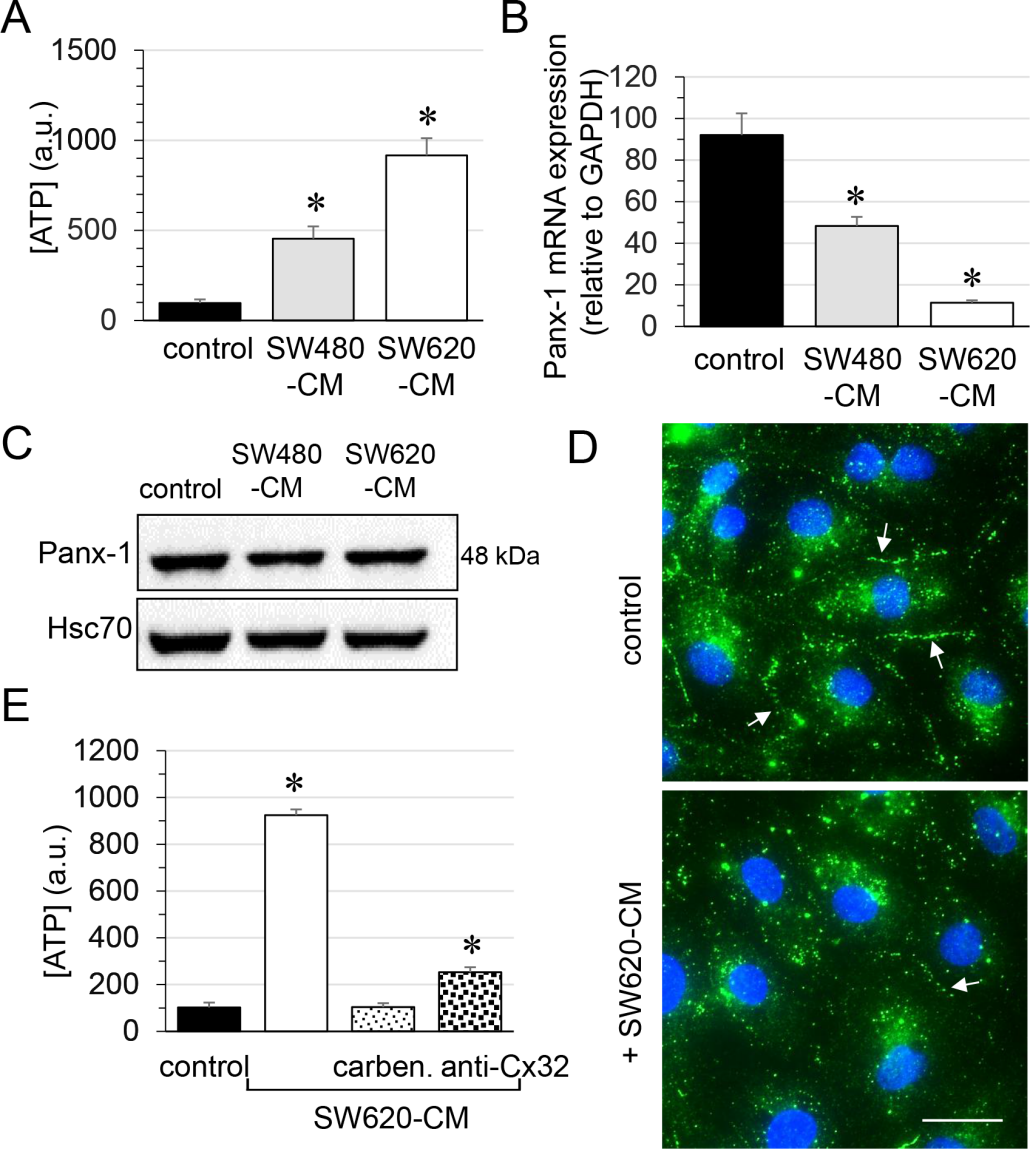
Role of endothelial Cx32 in SW620-CM-triggered ATP release in HMEC **A.** Both SW480 and SW620 cell-conditioned media triggered ATP accumulation in HMEC bath medium within 6 h. Extracellular ATP was measured by Luciferase assay (means ± S.D. *n* = 4; *P-values < 0.01 *vs* control). **B.** Panx-1 mRNA expression in HMEC after 6 h of control or CRC cell-CM exposures. A drastic decrease in Panx-1 expression was observed with SW620-CM (means ± S.D. *n* = 3; *P-values < 0.05 *vs* control). **C.** Panx-1 protein expression in HMEC was unchanged by exposure to SW480- and SW-620-CM for 6 h (mean ±SD, *P*-value*s* = 0.4980 Mann-Whitney U test; *n* = 4). **D.** Cell surface localization of Panx-1 was decreased in HMEC exposed to SW620-CM for 6 h. Arrows indicated Panx-1 plaques at the plasma membrane. DAPI staining of nuclei. Optical section of 0.5 μm thickness (*n* = 5, Bar 12 μm). **E.** SW620-CM-triggered ATP release is inhibited by gap junction blocker, carbenoxolone (carben., 100 μM, 30 min) and by neutralizing Cx32 antibody (0.2 μg anti-Cx32 mAb) in HMEC (means ± S.D. *P-values < 0.01 *vs* control; *n* = 3).

Since Cx32 was not involved in GJIC between cells (Fig. 1D and Suppl. Fig. S2B), we next explored the role of Cx32 hemi-channels in ATP release by endothelial cells [28, 29]. Incubation of endothelial cells for 6 hours with SW480-CM and SW620-CM induced a 5-fold and a 10-fold increase in ATP release, respectively (Fig. 5A). Although pannexin (Panx) channels have been involved in ATP release [30], these proteins might not be responsible for the observed effects as Panx-1 mRNA level dramatically decreased in HMECs exposed to SW480-CM and SW620-CM (Fig. 5B) while Panx-2 or Panx-3 were not expressed in HMECs in our culture conditions (not shown). Immunoblot analysis of Panx-1 in whole cell extracts of HMEC revealed no significant difference (*P* = 0.498) between control cells and those incubated with CRC-CM for 6 h (Fig. 5C). This may be explained by the long half-life of Panx-1 (more than 8 hours; [31]) which contrasts with the rapid turnover of Cx43 (with a short life-time of only 1-3 hours; [32, 33]). Nevertheless, cell surface localization of Panx-1 was strongly reduced by SW620-CM as seen by confocal microscopy (Fig. 5D). It is therefore unlikely that Panx-1 channels are responsible for the ATP release increased by SW620-CM. The gap junction blocker carbenoxolone completely blocked SW620-CM-induced ATP release, which was also dramatically reduced by the neutralizing anti-Cx32 mAb (Fig. 5E). Thus, one of the consequences of Cx32 expression increase in endothelial cells exposed to SW620-CM is the release of larger amounts of ATP.

### SW620 cell-secreted factors induce the endothelial Cx32 expression and tube formation via the cytokine receptor CXCR2

**Figure 6:**
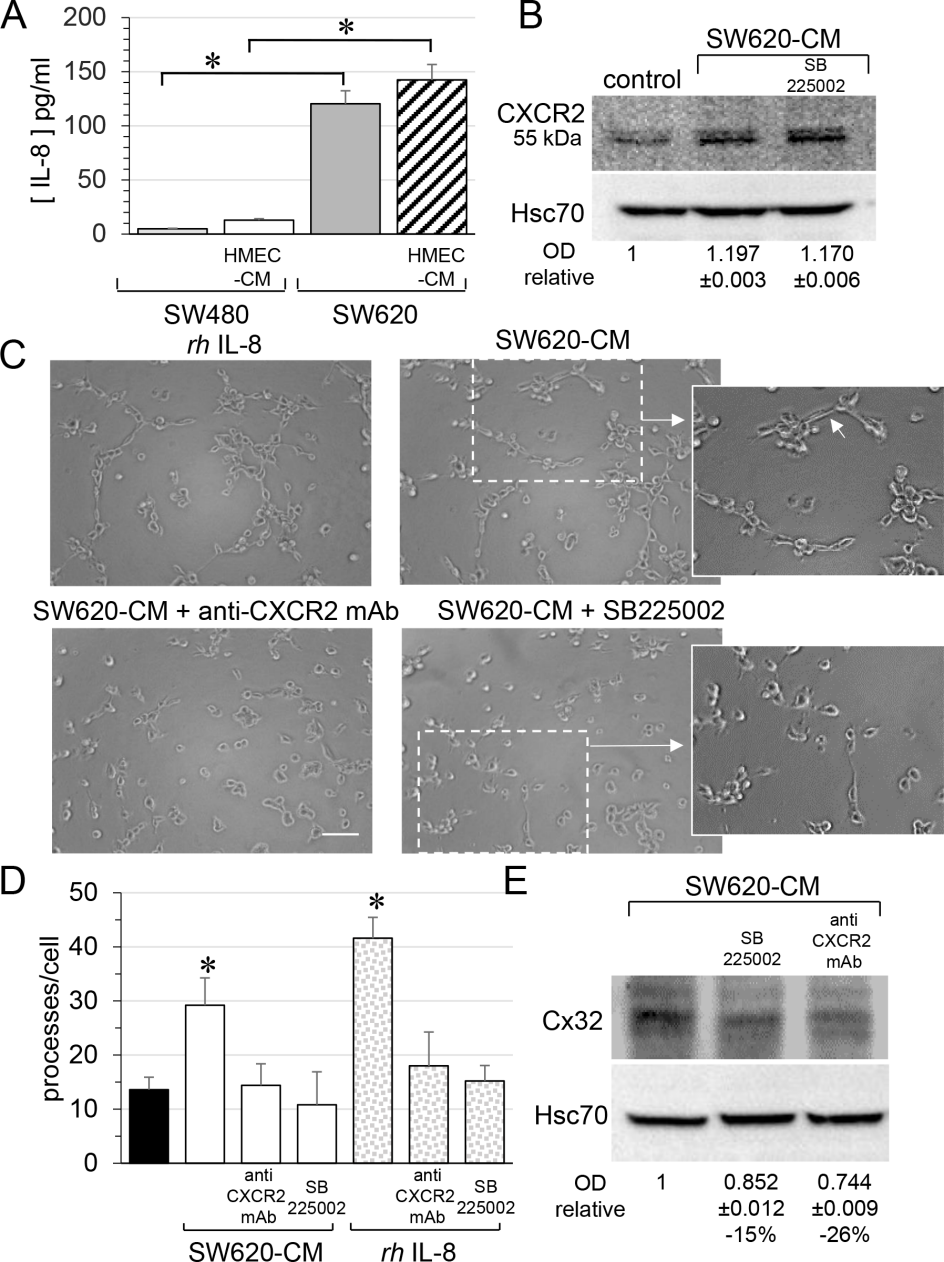
SW620 cell-secreted factors require CXCR2 signaling pathway to induce the endothelial Cx32 expression and tube formation **A.** IL-8 secretion in conditioned media from SW480 and SW620 cells was examined through ELISA. CRC cells were exposed or not to the HMEC-CM. All cell media were collected after 6 h (mean ± SD, *P-values < 0.01 Mann-Whitney U test and Kruskal-Wallis test; *n* = 4). **B.** SW620-CM increase the endothelial expression of the CXCR2 receptor. A slight but significant increase in optical density (OD; relative to control) of bands was detected in response to SW620-CM compared with unstimulated HMEC (*P*-values < 0.01 Mann-Whitney U test and Kruskal-Wallis test; *n* = 4). No inhibitory effect was observed by pre-treating HMEC with SB225002 (200 nM), the CXCR2 antagonist. Representative of 4 experiments (Hsc70 as loading control; 100 μg/lane). **C–D.** Endothelial CXCR2 conveys angiogenic effects of SW620-CM. HMEC were pretreated or not with neutralizing anti-CXCR2 antibody (anti-CXCR2 mAb; 10 μg/ml) or SB225002. Cells were exposed to SW620-CM or human recombinant *rh*IL-8 (1 ng/ml) for 6 h. C. Representative Images of tube formation (Bar 80 μm). The dotted areas are enlarged in the inserts on the right. Arrows indicated branch points. D. Number of branch points per field of view was quantified (mean ± SD, *n* = 4; *P < 0.01 *vs* control). **E.** Blocking CXCR2 significantly diminished SW620-CM-induced expression of Cx32 in HMEC (*P*-values < 0.01 *vs* SW620-CM Mann-Whitney U test and Kruskal-Wallis test; *n* = 3). HMEC were exposed to cell-conditioned media for 6 h. In some cases, HMEC were pretreated with anti-CXCR2 mAb or SB225002, as indicated. This is a representative of three experiments with similar results (Hsc70 as loading control; 100 μg/lane).

**Figure 7:**
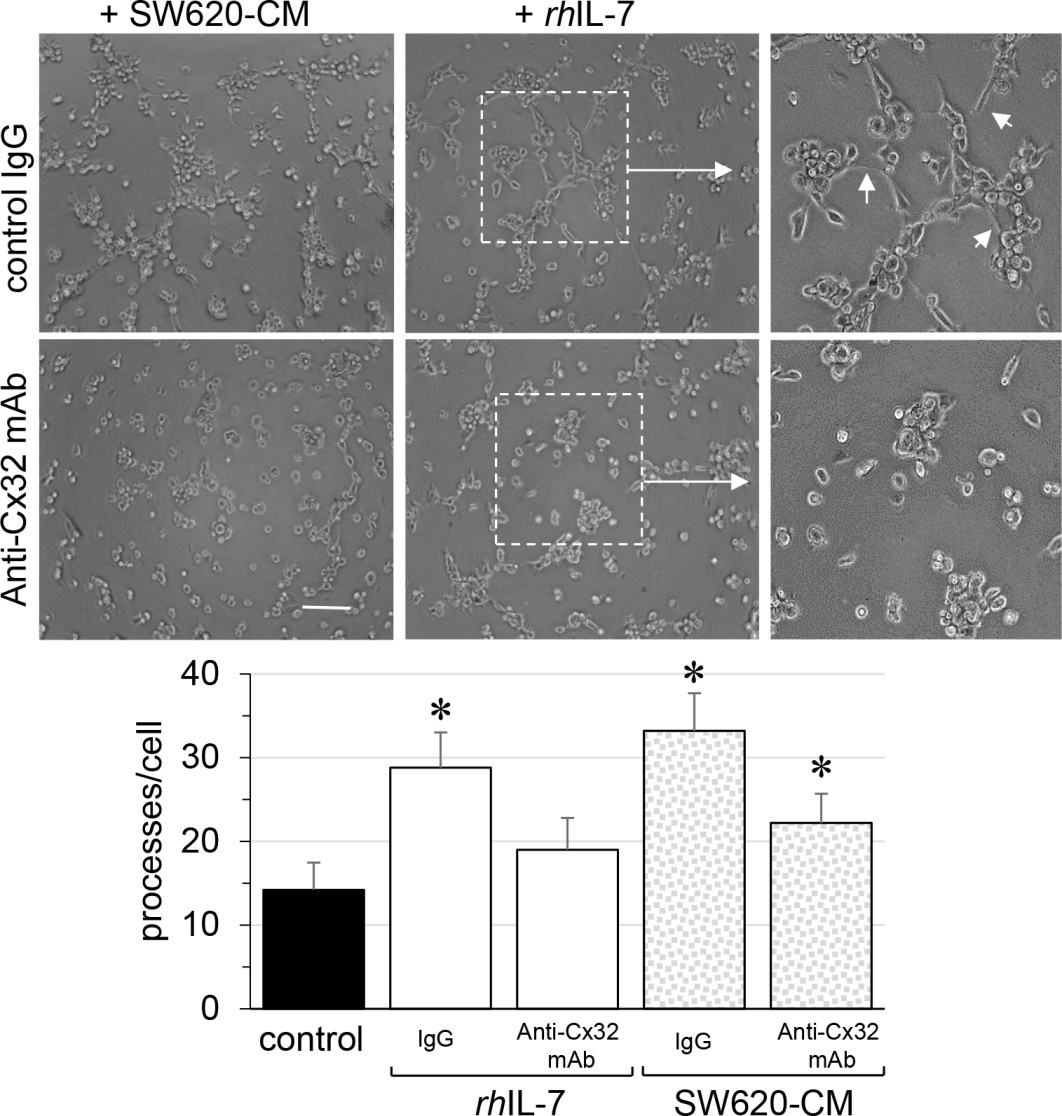
Endothelial Cx32 contributes to angiogenic effects of IL-7 as SW620-CM does *In vitro* tubulogenesis assay of HMEC pretreated or not (control IgG) with inhibitory monoclonal antibody against Cx32 (0.2 μg/ml anti-Cx32mAb). HMEC were plated on Matrigel-coated 24-well plates, incubated with SW620-CM or human recombinant *rh*IL-7 (1 ng/ml) for 6 h, and photographed (Bar 80 μm). The dotted areas are enlarged in the inserts on the right. Arrows indicated branch points. Histogram shows the number of branch points per field of view (at least 80 single cells were scored; mean ± SD, *n* = 4; *P < 0.01 *vs* control). Blocking Cx32 decreases *rh*IL-7- and SW620-CM-induced tube formation (*P < 0.05 *vs* control; *n* = 3).

Looking for the secreted factor that may account for the ability of SW620-CM to promote Cx32 expression in endothelial cells, we explored the production of interleukin-8 (IL-8) as metastastic tumor cells can release high levels of this cytokine [34, 35]. Accordingly, SW620 secreted much more IL-8 than SW480 cells, a secretion that was only slightly increased by incubation with HMEC-CM (Fig. 6A). Since IL-8 interacts with the G-protein-coupled receptor CXCR1 and CXCR2, we explored the expression of these receptors in endothelial cells. Unstimulated HMEC expressed no CXCR1 (not shown; [36]) and low levels of CXCR2 (Fig. 6B). After a 6 hour exposure to SW620-CM, CXCR1 remained undetected (not shown) whereas the expression of CXCR2 was increased (Fig. 6B). Pre-incubation of HMEC with a neutralizing anti-CXCR2 antibody or the CXCR2 inhibitor SB225002 [37] abolished the ability of SW620-CM (Fig. 6C) or IL-8 (Fig. 6D) to promote the formation of tubes by endothelial cells. Both the neutralizing anti-CXCR2 antibody and the CXCR2 inhibitor SB225002 attenuated Cx32 expression induced in HMEC by incubation with SW620-CM (Fig. 6E). In addition to IL-8, we show for the first time that IL-7 could be a potent angiogenic factor that induces tubulogenesis in a Cx32-dependent manner (Fig. 7). Pre-incubation with the Cx32 blocking antibody abolished the ability of IL-7 to promote the tube formation by HMEC. Altogether, SW620-CM induces both CXCR2 expression and function which promotes tubulogenesis, at least in part in a Cx32-dependent manner.

## DISCUSSION

**Figure 8:**
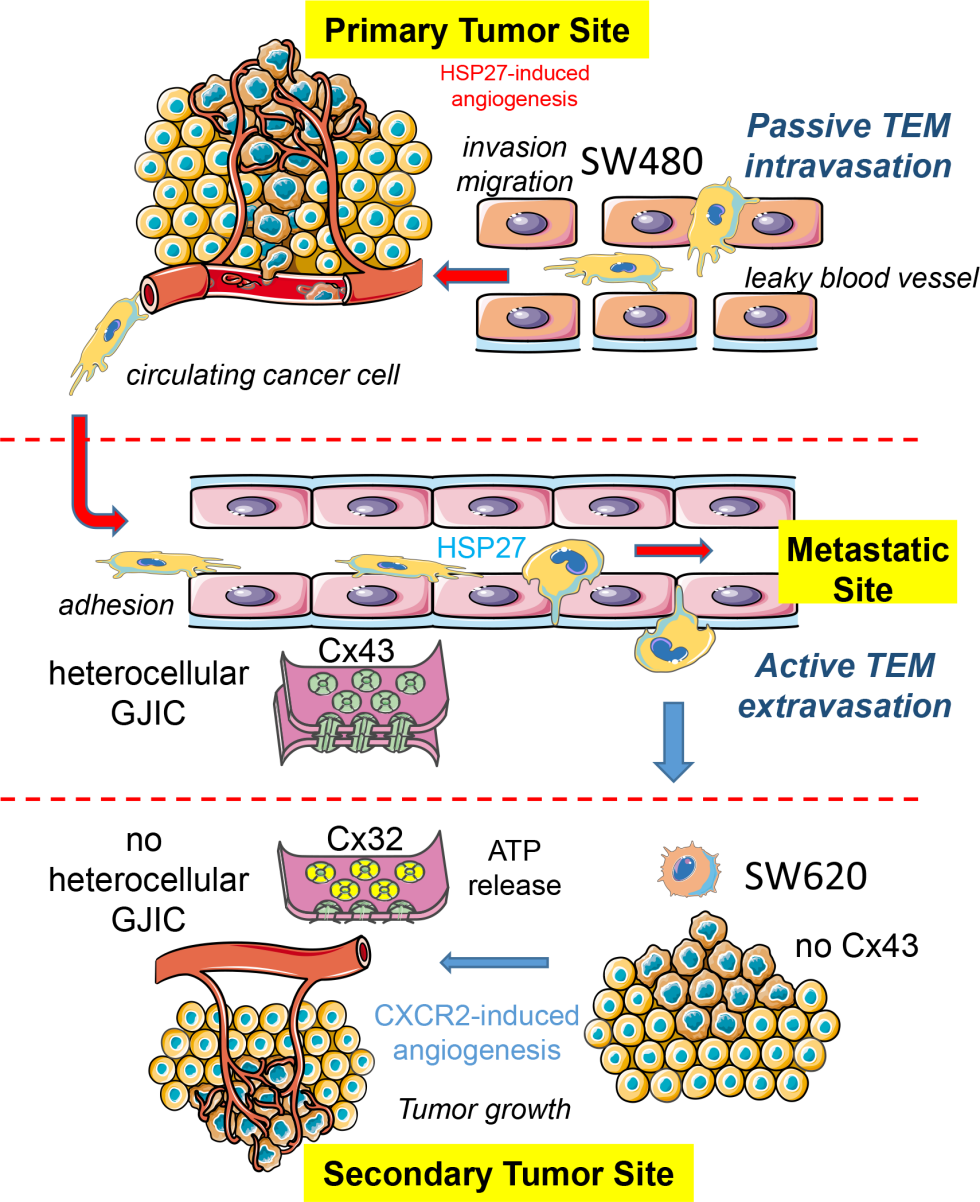
Hypothetical model of the endothelial connexin contribution to the colorectal cancer (CRC) pathogenesis The diagram shows the endothelial cell (EC) expression of both Cx32 and Cx43 as well as their ability to form hemi-channels or gap junction channels with CRC cells at the microvascular level. Cancer cells from a primary tumor (here, SW480 cells) locally invade the surrounding tissue, enter the microvasculature of the blood system (passive intravasation), survive and translocate through the bloodstream to micro-vessels of distant tissues. SW480 cells release HSP27 that favors the establishment of GJIC, via Cx43-channels, with the underlying endothelium. This direct cell-to-cell communication contributes to their trans-endothelial migration TEM (active extravasation). In contrast, cancer cells from a metastatic site (here, SW620 cells) release larger amount of chemokines, increasing the endothelial expression of the receptor CXCR2. In turn, CXCR2 promotes both endothelial Cx32 expression and tubulogenesis. The release of ATP through Cx32 hemi-channels from ECs and the subsequent ATP-mediated activation of purinergic P2Y2 receptors could modulate crosstalk between ECs and metastatic cancer cells, favoring neo-angiogenesis in metastatic foci.

The gradual loss of functional Cx43 gap junction and the increased expression of Cx32 in colorectal cancer biopsy were previously associated with a worst tumor grading, suggesting a role for these connexins in metastasis formation [11–13]. Here, we demonstrate that tumor cells can affect the expression of Cx proteins in endothelial cells. Cells derived from a primary tumor secrete high levels of HSP27 that promotes the phosphorylation of Cx43 in endothelial cells and the formation of gap junction between tumor and endothelial cells. Cells derived from a metastatic site in the same patient do not modulate Cx43 in endothelial cells but rather promote Cx32 expression and tube formation through a mechanism that involves CXCR2 expression. A model is proposed in Fig. 8.

A unique feature of SW480 and SW620 colon carcinoma cell lines is that they derive from primary and secondary tumors resected from the same patient [15], thus may represent a valuable resource for examining changes late in colon cancer progression [14, 38]. SW480 cells migrate faster than SW620 cells across HMEC monolayers, which is in agreement with their higher locomotion activity [39] and their higher capacity to generate metastasis in a xenograft model [14]. SW480 cells had been shown to release HSP27 whereas SW620 cells did not [16, 17]. HSP27 overexpression has been inversely correlated to metastatic behavior of human colorectal carcinoma (CRC) cells [16, 40–42]. Here, we show that HSP27 released by SW480 cells modulates the phosphorylation of endothelial Cx43, thereby increasing GJIC between SW480 cells and endothelial cells. This heterocellular GJIC may be a necessary step for extravasation of CRC cells from the blood flow to the metastatic site. Several kinases, including protein kinase A, protein kinase C, Cdc2/Cyclin B1 kinase, casein kinase 1, MAP kinase and Src family kinases, were shown to phosphorylate serine residues at the C-terminus of Cx43 [43–45]. While the precise kinase involved in the SW480-CM-induced changes in the phosphorylation of Cx43 serine residues in HMEC was not identified, this event favors 14-3-3 binding to Cx43. Such a binding increases the incorporation of Cx43 multimers into existing gap junctional plaques [23], and facilitates Cx43 channel formation [24] and GJIC formation between cells. Of note, 14-3-3 protein overexpression also promotes lung cancer progression when combined with HSP27 overexpression [46].

In contrast to HSP27-mediated effects of SW480 cells on Cx43 expression, SW620 cell-secreted factors up-regulate the endothelial expression of Cx32 and enhance tube formation via a CXCR2, suggesting a promoting effect on angiogenesis and, consequently, tumor growth [27]. It remains unknown if a common regulatory mechanism accounts for Cx32 overexpression and Cx43 down-regulation in endothelial cells. A cellular redistribution of Cx32 and Cx43 has been previously associated with the metastasis potential of CRC [11] and breast cancer [9] cells. We show that, by opening Cx32 hemichannels, SW620-CM triggers ATP release, which may not depend on P2X7 receptor activation or pannexin channels that can also release ATP in other cell settings. By activating specific purinergic receptors, ATP released through Cx32 hemichannels could modulate the crosstalk between cancer and endothelial cells, as do P2Y2 receptors in breast cancer metastasis [47].

CXCR2 is another critical component of tumor cell behavior and its expression in endothelial cells favors tumor angiogenesis [48]. In colorectal tumors, CXCR2 was identified on tumor cells, endothelial cells, infiltrating neutrophils, and macrophages [49, 50], and CXCR2 overexpression was identified in CRC liver metastases [51]. CXCR2 promotes tumor growth through recruiting pro-tumorigenic neutrophils and stimulating angiogenesis [52, 53]. A CXCR2 antagonist inhibits proliferation and invasion of CRC cells in an *in vitro* assays and the growth of tumor xenografts in immune-deficient mice [54]. CXCR2 can be activated in an autocrine-dependent manner [55], through one or several of its ligands (i.e. IL-1, 2, and 3, epithelial cell derived neutrophil-activating peptide-78/IL-5, granulocyte chemotactic protein-2/IL-6, IL-7, and IL-8). In addition to IL-8, extensively studied in *in vitro* and *in vivo* CRC cell models [34–36], we show here that IL-7 could be also a potent angiogenic factor that induces tubulogenesis in a Cx32-dependent manner. In breast cancer, IL-7 stimulates invasion and secretion of the lymphangiogenic factors VEGF-C and VEGF-D [56, 57].

To conclude, the differential ability of SW480 and SW620 cells to promote the expression and activation of Cx43 and Cx32, respectively, illustrates the functional heterogeneity of tumor cells in a given patient. Some tumor cells induce the formation of heterocellular GJIC via phosphorylation of Cx43 whereas other promote tubulogenesis via the induction of Cx32 expression. These tumor cells modulate their microenvironment through the release of soluble factors such as soluble HSP27. Further exploration of CRC cell-mediated endothelial junction remodeling may suggest novel approaches for blocking cancer cell migration and metastasis formation.

## MATERIALS AND METHODS

### Cells

Human microvascular endothelial cells (HMEC; Lonza; Basel, Switzerland) were grown in DMEM plus 10% FCS (5% CO_2_; 37°C). Human colorectal cancer cell lines, SW480 (ATCC CCL-228) and SW620 (ATCC CCL-227) were plated in DMEM plus 10% FCS. Untouched cell lines and HMEC were transfected by lipofectamine RNAiMAX (Invitrogen; Life Technologies, Saint-Aubin, Fr). siRNA HSP27 was purchased from Sigma-Aldrich (SASI_Hs01_00051449; Saint-Quentin Fallavier, Fr) and control siRNA was from Dharmacon (Fermentas; ThermoFischer, Saint-Remy-les-Chevreuses, Fr). Cells were incubated overnight in FCS-free media before use.

### Reagents

Low endotoxin rhHSP27 was purchased from Enzo Life Sciences (Villeurbanne, Fr) and rabbit anti-HSP27 from ABR (AffinityBioReagent, ThermoFisher, Fr). Recombinant human *rh*IL-8 and *rh*IL-7 were from R&D Systems. Mouse anti-Hsc70 was from Santa Cruz Biotech. Polymyxin B was from InvivoGen (Toulouse, Fr). Rabbit polyclonal anti-Cx43 (710700), mouse monoclonal anti-Cx43 (CX-1B1), anti-Cx32 (CX-2C2) and ZO-1 (ZO1-1A12) antibodies were from Invitrogen. Rabbit oligoclonal anti-Panx-1 (11HCLC) and rabbit polyclonal anti-CIP75 were from ThermoScientific (Rockford, USA) and anti-14-3-3, anti-phosphoserine and anti-CXCR2 from Abcam. DiL-C18, thapsigargin and fura-2/AM were from Molecular Probes. Other chemicals were from Sigma-Aldrich.

### Specific cell treatments

To avoid endotoxin contamination of rhHSP27, cells were preincubated with polymyxin B (PMB, 10 μM; 30-60 min) and rhHSP27 solutions were also treated with PMB prior to their use. To block TLR3, HMEC were pre-incubated with the neutralizing anti-hTLR3 (antiTLR3 mAb; 20 μg/ml) for 1 h (eBioscience, San Diego, CA, USA). To block the signaling of TLR2 and TLR4 induced by LPS, OxPAPC (30 μg/ml) was added (InvivoGen). To block CXCR2, HMEC were pretreated with 200 nM SB225002 (CXCR2 antagonist) for 30 min or with 10 μg/ml of neutralizing anti-CXCR-2/IL-8 RD (Clone 48311) for 1 h (R&D Systems). For collection of conditioned media (CM), confluent cells were grown overnight in FCS-free DMEM then fresh medium (4 ml/T-75 flask) was added for 6 h before to be collected.

### Fluorescence recovery after photobleaching (FRAP)

The GJIC between HMEC was measured by means of gap-FRAP method [18]. Cells were loaded with 10 ng/μl of calcein/AM for 15 min. The fluorescence of investigated cells was bleached at 405 nm. The recovery of fluorescence was measured at 488 nm every 20 sec for a time period of 8 min. The fluorescence in one unbleached cell was used to correct the artefact loss of fluorescence. The permeability of gap junctions is estimated by the diffusion rate constant *k* (expressed in min^−1^) determined from recovery curves as following: (Fi - Ft)/(Fi - F0) = e^−kt^, where Fi, Ft and F0 are intensities before bleaching, at time t and *t* = 0 respectively.

### Transendothelial migration (TEM) assay

HMEC were cultured on 8-μm membrane pores of Transwell inserts in 24-well plates until confluency (CytoSelectTM, Cell Biolabs; Euromedex, Mundolsheim, Fr). CRC cells were seeded on the top of HMEC monolayer (300,000 cells per well). After coculturing for 6 h, invasive cells on the membrane bottom were stained and quantified at OD 560 nm after extraction. Each experiment used triplicate wells and the same assay was repeated four time.

### Heterocellular GJIC functionality

CRC cells were labeled with 4 μM calcein/AM (30 min) together with 10 μM DiL-C18 as previously detailed [18]. After washing, 10^3^ fluorescent cells were laid on HMEC monolayers. The transfer of dye was visualized after a given time at 37°C.

### Immunodetection of protein phosphorylation

Cell were washed 5 times with ice-cold PBS and lysed (45 min on ice) using 1 ml of lysis buffer containing: 50 mM Tris-HCl, pH 7.4, 1% Nonidet P-40, 0.25% sodium deoxycholate, 150 mM NaCl, 1 mM EGTA, the phosphatase inhibitor cocktails 2 and 3 (1:100; Sigma-Aldrich) and protease inhibitor mixture (Roche Molecular Biochemical). Cells were scraped, centrifugated, and lysates collected. Proteins were boiled for 5 min in 2 μl SDS buffer, fractionated using 10% SDS-PAGE, and transferred to nitrocellulose membranes (Bio-Rad, CA). Membranes were blocked for 1 h with 5% BSA in Tris buffered saline with Tween 20 (0.1%) (TBS-T), and incubated overnight with antibodies.

### Immunoprecipitation

Briefly, cells were lysed in RIPA buffer, and immunoprecipitation was performed with antibodies, as previously described [19].

### Immunofluorescence and imaging

Cells were fixed in 4% PAF and permeabilized with 0.1% Triton X-100 [19]. Images were performed using a Leica SP2 RS confocal microscope (Z-series of 0.6 μm-optical sections; 512 × 512 pixels; Rueil-Malmaison, Fr). For co-localization, images were taken on Axio Imager 2 (Carl Zeiss GmbH) with an Apotome2 module (Optical sections of 0.5 μm; 512 × 512; Oberkochen, Germany).

### ELISA analyses

HSP27 levels in cell supernatants were evaluated using enzyme-linked immune-absorbent assay (ELISA kit; Enzo Life Sci. ADI-EKS-500) according to the manufacturer's instructions. The quantitative determinations of human IL-8 concentrations were made by enzyme-linked immunosorbent assays (ELISA Quantikine; R&D Systems) as previously described [19].

### Endothelial tube formation assay in collagen gels

HMEC were trypsinized and resuspended in ECM gel with DMEM or CRC cell's supernatants according to the manufacturer's instructions (from Cell Biolabs, Inc) [18]. For short term assays after 6 hours of incubation at 37°C, 80 single cells were scored for the number of processes per cell. Each well represents an n of 1 and is duplicated for each experiment, and each experiment was repeated three times. Cells were photographed at a magnification of x10 using Zeiss microscope, equipped with a video camera.

### Antibody transfer into HMEC

Inhibitory anti-Cx32 mAb (CX-2C2; Novex by Invitrogen) was transferred into HMEC using the PULSin protein delivery reagent according to the manufacturer's instructions (PolyPlus-transfection, New York, NY). Briefly, HMEC were grown to 70% confluence in 24-well tissue-culture plates and washed with PBS. A mixture of 20 μL of HEPES-buffered saline (HBS) with 0.2 μg anti-Cx32 mAb or control IgG (Sigma) and 0.8 μL PULSin was incubated at room temperature for 15 min. HMEC were incubated with 180 μL Opti-MEM with 20 μL antibody containing solution at 37°C for 4 h before used.

### ATP measurement

Concentration of ATP in cell media was detected by luciferin-luciferase assay (ENLITEN ATP Assay, Promega; Charbonnieres, Fr). HMEC were plated at 500 × 103 cells/cm 2, growth arrested in FCS-free medium and exposed to carbenoxolone (200 μM) or GAP26 (500 μM) and/or SW480- or SW620-CM. Supernatants were collected after 6 h, put on ice and centrifuged at 12,000 *g* for 10 min.

### Analysis of RNA expression for Panx-1

Total RNA extraction, first-strand DNA synthesis and semiquantitative reverse transcription-polymerase chain reaction (RT-PCR) were performed as described previously. The primer sequences are as follows: FP: CTGTGGACAAGATGGTCACG and RP: CAGCAGGATGTAGGGGAAAA.

### Statistical analysis

Results are expressed as mean ± SD. Groups were compared using one-way analysis of variance (ANOVA; Statview Software). Stimulated samples were compared to controls by two-tailed, unpaired *t*-tests. A Mann-Whitney *U* test was also used to compare data groups. In some cases, statistics were made with Tanagra software (http://freestatistics.altervista.org/?e_4) using a Kruskal-Wallis 1-way ANOVA. In all cases, *P values < 0.05 were significant.

## SUPPLEMENTARY MATERIALS AND METHODS


