# Supplementary material for: Primary tumor- and metastasis-derived colon cancer cells differently modulate connexin expression and function in human capillary endothelial cells
Source: Oncotarget. 2015 Aug 6;6(30):28800–15. doi: 10.18632/oncotarget.4894 (PMC4745693; doi:10.18632/oncotarget.4894)
Supplement: Supplementary file 1 [file oncotarget-06-28800-s001.pdf]

SUPPLEMENTARY MATERIALS AND METHODS

ALKALINE PHOSPHATASE TREATMENT

Alkaline phosphatase was used to dephosphorylate Cx43 immobilized on transfer membranes. Protein samples from cell lysats were separated by SDS-PAGE and transblotted onto membranes. A region of membrane corresponding to the migratory position of Cx43 was excised, placed in plastic containers containing 300 units/ml of activated alkaline phosphatase (Sigma) and incubated at 37°C for 4 h. Control membranes were similarly treated except without alkaline phosphatase. Following incubation, membranes were probed with polyclonal anti-Cx43 antibody (C-terminal).

UBIQUITINATION OF CX43

Confluent HMEC were treated or not with MG132 (10 µM; ready made solution from Sigma) for 4 h and coincubated with SW480-CM (collected after 6 h) for 0, 30 and 60 min. Cell lysates were immunoprecipitated with polyclonal anti-Cx43 antibody (C-terminal). Equal amounts of immunoprecipitates were subjected to SDS-PAGE, and ubiquitine was detected using anti-ubiquitine antibody (10H4L21; Pierce, ThermoScientific). Blots were stripped and reblotted with anti-Cx43 Ab.

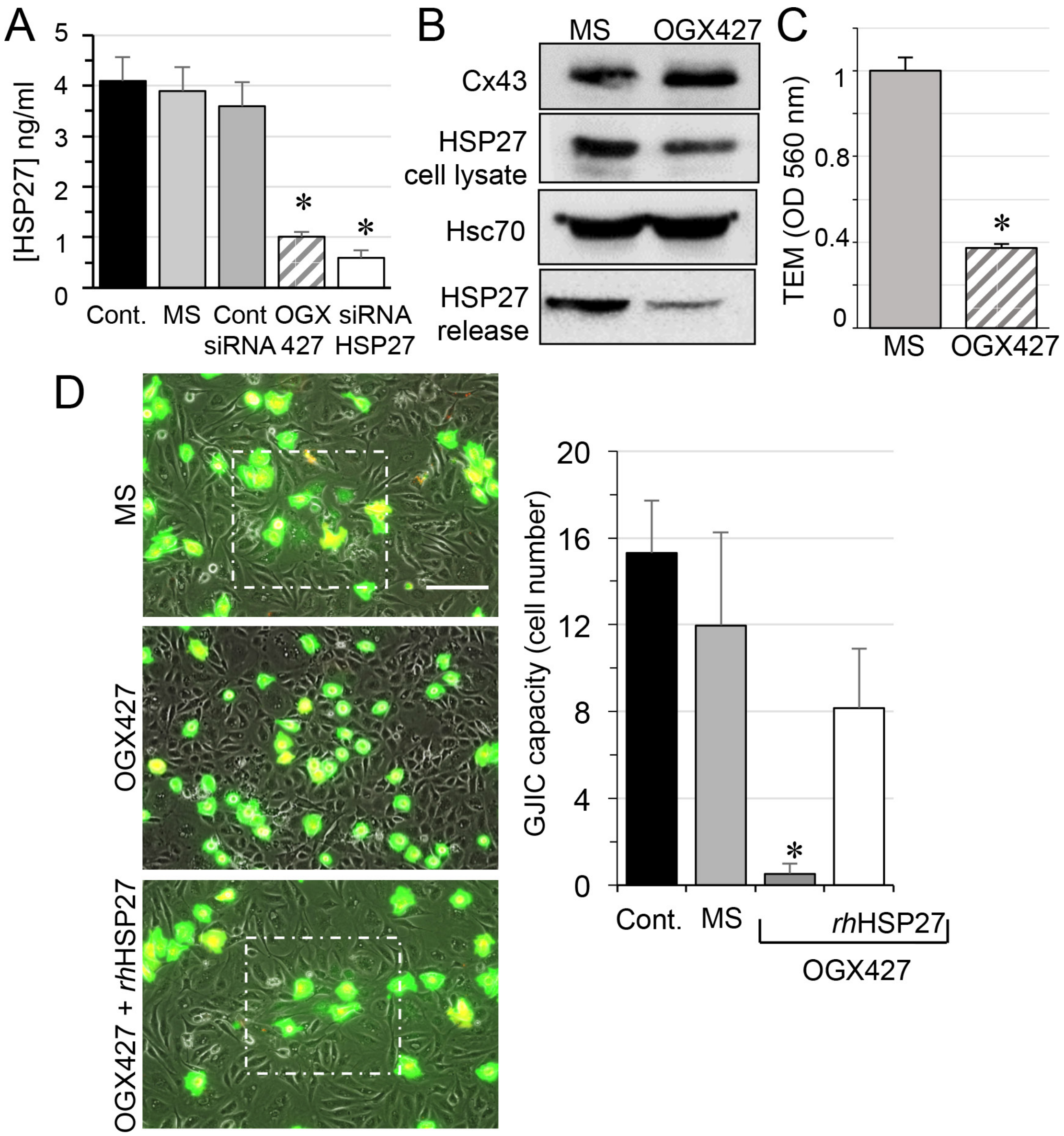

Supplementary Figure S1: HSP27 knockdown by treatment with OGX427 of SW480 cells inhibits their GJIC with HMEC. The sequence of the 2'-O-(2-methoxyethyl) antisense oligonucleotide against HSP27 (OGX-427; Oncogenex Tech. Vancouver, Canada) corresponds to the human HSP27 translation initiation site (5′-GGGACGCGGCGCTCGGTCAT-3'). An MS (5'-CAGCGCTGACAACAGTTTCAT-3′) was used as control (MS). **A.** HSP27 release by SW480 cells. Cells were transfected with HSP27 siRNA or OGX427, and their respective controls (Cont. siRNA or MS), 48 h prior to analysis, and compared to non-transfected cells (Cont.). Values are amounts of HSP27 measured by ELISA in supernatant of cells for 12 h (mean ± S.D., *$P < 0.05$ *vs* Cont.; $n = 4$). **B.** OGX-427 decreased HSP27 expression in SW480 cells. Representative immunoblot of HSP27 protein level in SW480 cells transfected with control MS and OGX-427 at 500 nM for 2 days (mean ± S.D., *$P < 0.05$; $n = 3$). **C.** OGX-427 slowed down the transendothelial migration of SW480 cells through HMEC monolayers. MS control and OGX-427 (500 nM) ($n = 3$). **D.** Inhibition of the heterocellular GJIC by OGX-427 was antagonised by the concomitant addition of 5 µg/ml rhHSP27. Note the morphological change of SW480 following treatment by OGX (mean ± SD, *$P < 0.05$; $n = 5$).

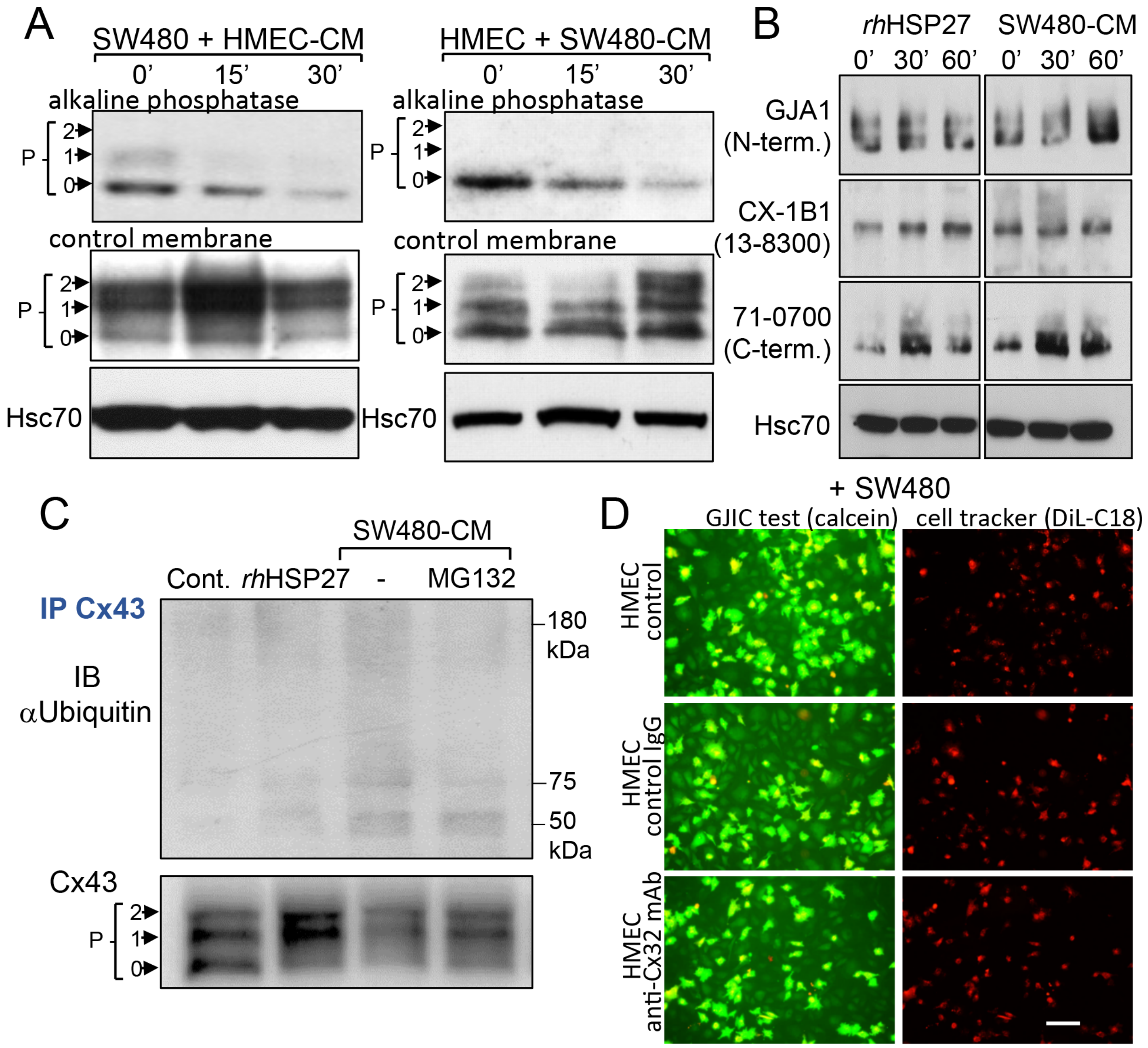

Supplementary Figure S2: Phosphorylation status of Cx43 in HMEC and SW480 cells. A. Effect of alkaline phosphatase treatment on immunorecognition of Cx43 in SW480 cells (right) and HMEC (left), respectively exposed to HMEC-CM and SW480-CM (collected after 6 h) for 0, 15 and 30 min. Following transblotting, membranes were incubated with alkaline phosphatase and probed with polyclonal anti-Cx43 Ab (C-terminal). **B.** Immunoblots showing selective recognition of the phosphorylation status of Cx43 in HMEC exposed to SW480-CM (after 6 h) or *rh*HSP27 (5 µg/ml) for 0, 30 and 60 min ($n = 3$). Cx43 was probed with the monoclonal Ab 13–8300 to detect its dephosphorylation (41 kDa; aa 360–376) or with polyclonal Abs, GJA1 (N-terminal, aa 114–144) and 71–0700 (C-terminal, aa 241–260). **C.** SW480-CM does not induce ubiquitination of Cx43. HMEC were pretreated or not with MG132 (10 µM), then exposed to SW480-CM, Control or rhHSP27 (5 µg/ml) for 60 min. Cell lysates were immunoprecipitated with polyclonal anti-Cx43 Ab (C-term.). Ubiquitin was detected by Western blotting. The blot was reprobed with the anti-Cx43 Ab. Representative of 3 experiments. **D.** Endothelial Cx32 is not involved in GJIC between SW480 and HMEC, pretreated or not with control IgG or the blocking anti-Cx32 mAb. SW480 (donors) preloaded with calcein/AM and labeled with DiL-C18, were plated with HMEC monolayer (receivers). HMEC establishing GJIC with SW480 become fluorescent by calcein diffusion. Microphotographs after 6 h of coculture ($n = 5$; Bar 100 µm).
